# Policy Coherence in US Tobacco Control: Beyond FDA Regulation

**DOI:** 10.1371/journal.pmed.1000079

**Published:** 2009-05-19

**Authors:** Joshua S. Yang, Thomas E. Novotny

**Affiliations:** 1 Center for Tobacco Control Research and Education, University of California San Francisco, San Francisco, California , United States of America; 2 Graduate School of Public Health, San Diego State University, San Diego, California, United States of America

## Abstract

Joshua Yang and Thomas Novotny explore whether the US government can develop and implement a coherent policy agenda to reduce tobacco-related morbidity and mortality.

## Introduction

As the Obama administration moves to enact meaningful and comprehensive health care reform in the United States, tobacco control must be elevated as a public health priority [1]. Though tobacco control efforts have been recognized as a top public health achievement of the 20th century [2], tobacco use continues to be the leading preventable cause of death in the US [3]. As Box 1 shows, the US bears a heavy burden from the health and fiscal effects of smoking. Thus, continued progress in preventing tobacco use and promoting smoking cessation must be a leading priority for health care reform under the new administration. This policy paper gives the current status of tobacco control policies, initiatives, and legislative action at the time of going to press.

**Box 1. Health and Economic Burdens of Smoking, United States**

**Tobacco use…**

**…is the leading preventable cause of death**. At least 443,000 annual premature deaths in the United States from 2000–2004 were attributable to smoking [33].

**…leads to premature death.** During 2000–2004, 5.1 million years of productive life were lost due to cigarette smoking and exposure to passive smoking per year [33].

**…contributes to health disparities.** African Americans, Native Americans/Alaska Natives, the poor, and people with lower educational attainment suffer from a higher burden of disease and disability from smoking.

**…is a major cause of cancer in the lung, larynx, pharynx, mouth, and bladder.** It also causes cancer in the pancreas, cervix, kidney, and stomach.

**…causes deaths from heart disease, stroke, and chronic obstructive pulmonary disease.**

**…is a fiscal burden.** Cigarette smoking and exposure to tobacco smoke results in productivity losses of $96.8 billion annually [33] and over $75 billion in annual US medical expenditures [6].

A bill to grant the US Food and Drug Administration (FDA) regulatory authority over tobacco products [4] was the central element of federal tobacco control efforts during the Bush administration. The bill, recently passed by the US House of Representatives, was drafted to obtain approval from a Republican Congress and the Bush White House. With a new administration in place and broad political and public support for health care reform, however, a comprehensive reassessment of the federal agenda on tobacco control is needed. Efforts to pass strong legislation to grant FDA regulatory authority over tobacco products must continue, but can no longer be the central focus of tobacco control efforts at the federal level.

This paper explores the potential of the US government (USG), with its extraordinary reach and extensive infrastructure, to develop and implement a policy-coherent agenda—defined as a series of consistent and mutually supportive institutional approaches to an important public health problem—to reduce tobacco-related morbidity and mortality. Tobacco use prevention and cessation measures have public support from both nonsmokers and smokers; in fact, 70% of smokers desperately want to quit [5]. These measures also mitigate health care costs [6]. To fully realize these cost savings and to answer the public's support for tobacco control measures, however, a functional approach requires policy integration across agencies, especially those under the Chief Executive, and support from the legislative branch of government.

## Prioritizing Health

Over the past half-century, health has occupied a prominent role on the policy agenda for many US presidents, with some administrations having made attempts to achieve policy coherence on specific health issues. For example, President Johnson identified reducing heart disease, cancer, and stroke as a national health priority [7], and President Nixon initiated a “War on Cancer” [8]. President Clinton coordinated federal action on HIV/AIDS, an action built on by President George W. Bush's President's Emergency Plan for AIDS Relief (PEPFAR) program [9].

In spite of successive surgeon general's reports and the recent recommendations from the Institute of Medicine (IOM) [10] and the President's Cancer Panel [11], comprehensive and concerted national action to reduce the burden of tobacco use has not been evident. What has been lacking is forceful and committed leadership from both the Office of the President and the US Congress. Yet none of the recent reports that offer national plans to end the tobacco epidemic fully appreciate the numerous federal agencies that have a role to play in a policy-coherent federal tobacco control agenda. The surgeon general's 2000 report emphasized the role of state governments [12], and the IOM's report (“Ending the Tobacco Problem: A Blueprint for the Nation”) urged strong Congressional action [10], while the President's Cancer Panel extensively covered broad federal actions [11]. We focus on the breadth of USG departments and agencies to examine how strong leadership from the Office of the President can marshal the considerable resources of many agencies in order to reduce the burden of tobacco use in the US.

## Federal Policies and Programs in Tobacco Control

USG agencies cover a range of populations, environments, products, and functions that may effectively support tobacco control. However, they are uncoordinated across agencies and not sufficiently on-message to be considered a coherent national strategy. To understand possible future federal action, we first describe briefly the roles of the current most important USG agencies in tobacco control. A more detailed discussion of existing policies and programs can be found in Text S1.

### Department of Health and Human Services (DHHS)

DHHS is the lead department for current tobacco control activities. The potential of the department to significantly reduce the level of smoking and the burden from tobacco-related diseases through its vast infrastructure is enormous, but DHHS has faced a number of barriers in this role. Policy coherence in tobacco control across the federal government begins with mobilization of the resources and infrastructure within DHHS.

The Centers for Disease Control and Prevention (CDC), one of 12 agencies within DHHS, houses the National Tobacco Control Program under the Office on Smoking and Health (OSH). It provides technical assistance to states and engages in programmatic activities while the National Institutes of Health (NIH), mainly through the National Cancer Institute, National Institute on Drug Abuse, and the Fogarty International Center, is the lead agency for tobacco control research. Both CDC and NIH also play critical tobacco use surveillance roles [13]. The NIH Tobacco and Nicotine Research Interest Group (TANRIG) was formed in January 2003 with the goal of increasing collaboration, coordination, and communication of tobacco- and nicotine-related research among NIH institutes and centers, and with partnering DHHS agencies.

Smoking cessation services are provided to specific populations through various DHHS programs, such as Medicare and Medicaid, the public health insurance programs for the elderly and the poor, respectively [14]. The coverage of these programs, however, is insufficient and needs to be strengthened [15]. Other DHHS agencies have a variety of concerns and jurisdictions relative to tobacco control. For example, the Agency for Healthcare Research and Quality [16], CDC [17], and the US Public Health Service [18] produce important best practices guidelines for smoking cessation. The Substance Abuse and Mental Health Services Administration (SAMHSA) has a mandate to enforce a state minimum tobacco product purchase age [19].

### Non-Health Agency Tobacco-Related Concerns

The Federal Trade Commission, a consumer protection and fair competition agency within the USG, oversees cigarette package warning labels [20] and has broad authority over tobacco product marketing and advertising, collects data on the marketing expenditures, and conducts tests to assess the tar and nicotine levels of cigarettes.

With the creation of the Department of Homeland Security, responsibility for the collection and enforcement of tobacco excise taxes has been split between the Department of The Treasury and the Department of Justice (DOJ). The Treasury department, through the Alcohol and Tobacco Tax and Trade Bureau, is responsible for the collection of tobacco excise taxes. The Bureau of Alcohol, Tobacco, Firearms and Explosives within the DOJ is responsible for monitoring and eliminating smuggling of tobacco within and to the US.

Department of Veterans Affairs (VA), responsible for providing services to former soldiers, supports smoking cessation through its health facilities.

## Tobacco Control Priorities at the Federal Level

Three future tobacco control issues should be prioritized within the federal government: ratification of the World Health Organization (WHO) Framework Convention on Tobacco Control (FCTC), authorizing the FDA to regulate tobacco products, and settling the DOJ's Racketeer Influenced and Corrupt Organizations Act (RICO) case against the tobacco industry. Each of these issues should be part of a larger policy-coherent plan for the federal government on tobacco control, but are highlighted here for their significance and potential impact.

The FCTC is the first-ever global public health treaty [21]. It was developed to counter the globalization of tobacco use and the growing burden of disease from tobacco use worldwide. Though it was signed by then-DHHS Secretary Tommy Thompson, President Bush did not send it to the Senate for ratification. Ratification of the FCTC should be a leading priority within health reform for the Obama administration. It will provide the needed impetus to revive comprehensive tobacco control efforts at the national level and act as a framework for developing a national tobacco control agenda.

There is little dispute over whether the FDA should regulate tobacco products; there is controversy, however, over whether the existing bill is the appropriate legislation to grant that authority [22],[23]. Granting FDA authority over tobacco products should be a high priority within health reform. The Obama administration should work to ensure that legislation contains the strongest possible language and conditions beneficial to public health without concessions to the tobacco industry.

Federal Judge Gladys Kessler found the tobacco industry defrauded the American public and violated two sections of the RICO Act in the DOJ's case against the tobacco industry, *United States v. Philip Morris* [24]. Though the case is currently in appeal, Judge Kessler's remedies for corrective action order the tobacco industry to cease false and deceptive activities. They also call for government regulatory authority over tobacco products and assertion of greater oversight of the industry through disclosure of industry documents and reporting of disaggregated market data.

Bolstering the smoking cessation capacity of the health care system will also be a tobacco control priority at the federal level if the Obama administration successfully advances its health care reform agenda. A commitment to prevention, long neglected within the US health system, is an essential component of health care reform in the US [1],[25]. Successful reform of the US health care system into a universally accessible, prevention-oriented system would include a strengthened infrastructure to support smoking cessation [26].

## Expanding USG Tobacco Control Efforts

The current financial and political environment makes it difficult for the USG to contemplate any new programs aside from those that revive the economy and seek a resolution to the conflicts in Iraq and Afghanistan. Tobacco control, however, is nonetheless a critical element of the domestic health care reform agenda for two reasons. First, it can be initiated at little to no cost to the government. Creating smoke-free environments and engaging in public education are simple first steps that require little more than asserting tobacco control as a public health priority. Programs and policies that require government expenditure, including increased provision of smoking cessation services, can be introduced as the country's economic condition stabilizes. Second, the economic return on investment in tobacco control could be remarkable [27],[28]. Thus, renewed commitment and mobilization within the programs and agencies described above, as well as others in the federal government, may be critical components of any health reform strategy for the new administration. The following agencies have a potentially important role to play in a revitalized national approach.

### Smoke-Free Environments

The Occupational Safety and Health Administration, Environmental Protection Agency, Department of Housing and Urban Development, Department of the Interior, and Department of Defense all have jurisdiction over spaces that can be made smoke-free.

### Smoking Cessation

All agencies that provide health services, and those employing federal employees, especially the VA, should provide easy access to comprehensive smoking cessation services [15].

### Public Education

CDC, NIH, and non-federal partners can collaboratively sponsor a national mass media counter-marketing campaign. The US Department of Education and US Department of Agriculture (USDA) also have public education roles.

### Research and Surveillance

For agencies like CDC and NIH to pursue tobacco control research and surveillance, oversight authority given to the Office of Management and Budget, the White House office responsible for overseeing the execution of the federal budget in executive branch agencies, to approve federal surveys and manage scientific information must be relaxed or removed [29].

### Product Regulation

Granting FDA regulatory authority over tobacco products is an essential part of a national tobacco program. Expanded regulation of tobacco as a crop and crop diversion programs by USDA are also needed [30].

### Industry Regulation

The DOJ must insist on strong remedies to the industry's behavior in the RICO case. In general, the USG should not obstruct litigation against tobacco companies and should allow judicial processes to play out in both individual and class action cases. Current regulation and surveillance of tobacco product sales, marketing, and promotion by the Federal Trade Commission and SAMHSA should be strengthened through FDA regulation of tobacco products or other legislative powers. US warning labels, for example, have been shown to have less impact than those in other countries [31].

### Foreign Policy on Tobacco and Health Trade Policy

The Doggett Amendment, which prohibits the Justice, State, and Commerce Departments from promoting the interests of the tobacco industry overseas (except in cases of discriminatory policies) [32], should be made into law. In trade agreements, the US Trade Representative should treat tobacco products as exceptional goods and, at minimum, be required to show that trade in tobacco products will not cause public health harm before being included in trade agreements and settlements.

### FCTC

The United States can demonstrate strong international leadership on tobacco control by ratifying the treaty and moving quickly to begin its implementation. Concerns over the treaty's implementation, including state compliance with FCTC binding obligations, are unwarranted under the FCTC's accommodations for differences in national governmental structure and laws.

The US can also provide foreign aid and technical assistance to support other countries in implementation of the FCTC. These efforts may involve NIH to support international research, CDC to support program implementation, USDA to support crop diversification, and USAID health programs to support development of health systems approaches to tobacco control.

## Summary and Conclusions

The wide public support and increasing political momentum for health care reform is an opportunity to advance the progress of tobacco control efforts, reducing the health toll on Americans and the economic burden on the American health care system. National plans to substantially reduce the burden of tobacco use have been put forth [10],[11]. Yet those plans are unlikely to be fully realized without the strong and committed leadership of the Office of the President and support of Congress. Existing policies and programs and opportunities for expansion of tobacco control within the federal government lead to the following recommendations for action.

### Presidential Leadership

President Obama should make a strong public commitment and mobilize the vast capacity of the USG to achieve policy coherence in tobacco control. This is especially true for mobilization of USG agencies to reorganize to facilitate interagency cooperation. An Executive lead organization should be designated to develop a national tobacco control plan. Coordination and leadership for a national tobacco control plan should be centralized in a highly visible and reputable lead agency, such as DHHS or the White House Office of Health Reform, with additional programmatic resources drawn from other agencies. Lead agency staff would coordinate programmatic activities with support from the White House. The Obama administration must also provide leadership through the budget process with Congress. Financial commitment from the US Congress and political support from the White House would facilitate participation from across agencies.

### Participation and Buy-In

A comprehensive national tobacco control plan would require broad-based participation and buy-in. The lead agency should work with partners including nongovernmental organizations, foundations, and professional organizations and societies to develop public support for the program. A broadly inclusive and neutrally facilitated process of stakeholder consensus building could be used to develop the central policies of a national tobacco control plan.

### Interaction with the States

Though the current network of state and local tobacco programs has benefited greatly from the leadership of the CDC–OSH National Tobacco Control Program, state and local governments are wary of federal legislative action on tobacco control. Concerns over preemption of state and local legislation by weak federal laws are bolstered by the use of preemption as an explicit strategy of the tobacco industry to subvert local tobacco control efforts. The actions of the federal government, adjusted to address concerns over preemption, should act to complement and bolster those of the states.

Though a vast majority of the success in tobacco control has been at the state and municipal level, federal involvement in tobacco control is needed for four reasons. First, some actions, such as product regulation and control of smuggling, are beyond the abilities of states. Second, the infrastructure of the federal government is so vast as to enable a coordinated and directed tobacco control program across states. Third, federal involvement can help to strengthen the tobacco control efforts of states with less effective programs. Last, areas such as foreign policy and the military fall solely within the purview of the federal government.

### Implications for Global Tobacco Control

As the implementation of the FCTC proceeds, countries will be implementing an array of tobacco control policies as part of their international commitment. The principle of policy coherence outlined in this paper for the US may spur countries to think more broadly across government functions about the ways in which the FCTC is implemented. Action toward policy coherence in the United States, with its substantial material and human capital resources, may act as a model for other countries to follow, including both its successes and its failures.

A comprehensive federal agenda on tobacco control will be a critical part of the Obama administration's health care reform efforts. After eight years of neglect on the leading cause of death in the United States and a prolonged legislative conundrum over tobacco products and nicotine regulation, a drastic policy change—one geared toward policy *coherence*—is in order. Most urgently, ratification of the FCTC, FDA regulatory authority over tobacco products, and a firm resolution to the RICO case must become reality. For these areas to be adequately addressed and carried out, however, USG agencies must be brought together and empowered to take concerted action on tobacco control that results in true policy coherence. We believe this change in direction is based on sound science, is acceptable to the almost 80% of nonsmoking Americans and the 70% of smoking Americans who want to quit, and in the best fiscal and health interests of the United States. It is change that we not only need, but is long overdue.

## Supporting Information

Text S1 Longer, more comprehensive version of the article.
(0.65 MB DOC)

## Abbreviations

CDC: Centers for Disease Control and Prevention
DHHS: Department of Health and Human Services
DOJ: Department of Justice
FCTC: Framework Convention on Tobacco Control
FDA: US Food and Drug Administration
IOM: Institute of Medicine
NIH: National Institutes of Health
OSH: Office on Smoking and Health
RICO: Racketeering Influence and Corrupt Organizations
SAMHSA: Substance Abuse and Mental Health Services Administration
USAID: US Agency for International Development
USDA: US Department of Agriculture
USG: United States Government
VA: US Department of Veterans Affairs
WHO: World Health Organization
