# Supplementary material for: Policy Coherence in US Tobacco Control: Beyond FDA Regulation
Source: PLoS Med. 2009 May 19;6(5):e1000079. doi: 10.1371/journal.pmed.1000079 (PMC2677110; doi:10.1371/journal.pmed.1000079)

**Policy coherence in U.S. tobacco control: Beyond FDA Regulation**

Joshua S. Yang, Ph.D., M.P.H.

(Corresponding author)

Center for Tobacco Control Research and Education

University of California, San Francisco

San Francisco, California

United States of America

Thomas E. Novotny, M.D., M.P.H.

Graduate School of Public Health
San Diego State University

San Diego, California

United States of America

Word Count: 10,479

As the Obama administration moves to enact meaningful and comprehensive health care reform, tobacco control must be elevated as a public health priority [1]. Though tobacco control efforts have been recognized as a top public health achievement of the 20^th^ century [2], tobacco use continues to be the leading preventable cause of death in the United States [3]. As Table 1 shows, the United States bears a heavy burden from the health and fiscal effects of smoking. Thus, continued progress in preventing tobacco use and promoting smoking cessation must be a leading priority for health care reform under the new Administration.

The Family Smoking Prevention and Tobacco Control Act [4], a bill drafted with hopes of approval from a Republican Congress and the Bush White House, was the central element of federal tobacco control efforts during the Bush Administration. The bill, passed by the U.S. House of Representatives in April 2009, would grant the U.S. Food and Drug Administration (FDA) authority to regulate tobacco products. It has overwhelming support from public health groups, with some criticism from others. The concerns include Philip Morris’s role in drafting the legislation, the inclusion of industry representatives as non-voting members on the oversight committee, limitations on FDA authority to ban classes of tobacco products, and the exclusion of menthol as a prohibited additive to cigarettes, among other issues [5,6]. With a new administration in place and broad political and public support for health care reform, however, a comprehensive reassessment of the federal agenda on tobacco control is needed. Efforts to pass strong legislation to grant FDA regulatory authority over tobacco products must continue, but cannot be the only focus of tobacco control efforts at the federal level.

This paper explores the potential of the Federal government, with its extraordinary reach and extensive infrastructure, to develop and implement a policy coherent agenda – defined as a series of consistent and mutually supportive institutional approaches to an important public health problem – to reduce tobacco-related morbidity and mortality. Tobacco prevention and cessation measures have public support from both non-smokers and smokers; in fact, 70% of smokers desperately want to quit [7]. They also mitigate health care costs [8]. To fully realize these cost savings and to answer the public’s support for tobacco control measures, however, requires policy integration across agencies, especially those under the Chief Executive, and support from the legislative branch of government.

# Prioritizing health

Over the past half-century, health has occupied a prominent role on the policy agendas for many U.S. Presidents. Though health is rarely the highest priority, some Administrations have made attempts to move toward policy coherence on some specific health issues. In February 1964, President Lyndon Johnson delivered his Special Health Message to Congress, identifying heart disease, cancer and stroke as the three health conditions to which the government would commit its resources [9]. In addition to fiscal support, the President formed the Commission on Heart Disease, Cancer and Stroke, which issued the report, *A National Program to Conquer Heart Disease, Cancer and Stroke* [9]. The Commission’s work led to the creation of Regional Medical Programs to foster collaboration among health service organizations in order to assure the availability of medical technologies to the public^1^.

In his 1971 State of the Union address, President Richard Nixon initiated a “War on Cancer” by requesting a special appropriation for $100 million to “launch an intensive campaign to find a cure for cancer” [10]. The initiative, conceived of as a coordinated and centralized effort of the Federal government, received full government backing with the signing of *The National Cancer Act* [11]. The Act created the National Cancer Program under the direction of the National Cancer Institute (NCI) and led to appropriations for $1.5 billion from 1971-1974. The infrastructure set up by the act supports continuing growth in cancer research funding. From 1997 to 2003, NCI’s budget grew from $2.381 billion to $4.592 billion, but has remained at that level since 2003 [12].

President Bill Clinton coordinated federal action on HIV/AIDS by establishing the National Office on AIDS Policy and the President’s Advisory Council on HIV and AIDS (PACHA). From 1995 to 2000, HIV/AIDS spending by the federal government rose from $6.7 billion to $10.8 billion, 0.6% of total spending in 2000 [13]. Under advisement from PACHA, President George W. Bush, in his 2003 State of the Union Address, announced the President’s Emergency Plan for AIDS Relief (PEPFAR), a 5-year, $15 billion dollar initiative to address HIV/AIDS, tuberculosis, and malaria in countries especially impacted by the disease. PEPFAR was approved by Congress and renewed in 2008, authorizing up to $48 billion to achieve the program’s goals [14].

President Clinton outlined key principles of national tobacco legislation [15], but tobacco control activities under his administration, including assertion of regulatory jurisdiction over tobacco products by the FDA and codification of the terms of the Master Settlement Agreement (MSA) through the 1997 McCain Bill, ultimately failed.

Successions of U.S. Surgeons General have attempted to create official policy on tobacco control through the Surgeon General’s reports on smoking and health and health promotion and disease prevention (now the Healthy People reports). The Surgeon General’s 2000 *Reducing Tobacco Use* provided a detailed overview of the central issues that must be addressed to reduce tobacco use [15]. More recently, the Institute of Medicine (IOM) report, *Ending the Tobacco Epidemic,* offered 42 recommendations for federal, state, and local government, health care providers, non-profit organizations, and other stakeholders to act as a blueprint to reduce smoking so substantially that it is no longer a public health problem in the United States [16]. Similarly, the President’s Cancer Panel has identified tobacco use prevention and treatment as a critical component of eliminating the burden of cancer in the United States, outlining recommendations for action for federal, state, and local governments, non-governmental organizations, the educational system, media, employers, insurance, and the health care system to reduce tobacco-caused deaths and disease [17].

In spite of these policy initiatives, comprehensive and concerted national action to reduce the burden of tobacco use has not been at the forefront of federal health policy. What has been lacking is forceful and committed leadership from both the Office of the President and the U.S. Congress in order to reduce the 443,000 annual premature deaths due to tobacco in the United States. Previous reports are notable in the lack of attention given to the vast reach of the U.S. Government (USG) to enact tobacco control policies across populations and environments. The Surgeon General’s 2000 report on reducing tobacco use focuses on the role of state governments. The IOM report focuses on Congressional actions, while the President’s Cancer Panel covers broad actions the federal government can take in reducing the burden of tobacco use. Yet none fully appreciate the numerous federal agencies that have a role to play in a policy coherent, federal tobacco control agenda. We focus on the breadth of USG departments and agencies to examine how strong leadership from the Office of the President can marshal the considerable resources of the USG across agencies to reduce the burden of tobacco use in the Untied States.

## **Federal policies and programs in tobacco control**

USG agencies cover a range of populations, environments, products and functions that may effectively support tobacco control. There are policies and programs on tobacco control throughout the federal government, but they are not coordinated effectively across agencies. Thus, these activities are not sufficiently on-message to be considered a coherent national strategy. To recommend possible future coherent federal action, we first describe briefly the roles of the current most important USG agencies in tobacco control.

Department of Health and Human Services (DHHS)

DHHS is the lead department for current tobacco control activities. The scope of the infrastructure and programs administered by DHHS make it the government agency that touches the lives of more Americans than any other. DHHS agencies provide services to special populations, including families and children (Administration for Families and Children), the elderly (Medicare), the poor (Medicaid), Native American/Alaska Natives (Indian Health Service [IHS]), as well as those that cover the entire population, including food and drug safety (FDA), public health (Centers for Disease Control and Prevention [CDC]), health care (Agency for Healthcare Research and Quality [AHRQ], Health Resources and Services Administration), and medical research (National Institutes of Health [NIH]). The potential of DHHS to significantly reduce the level of smoking and the disease burden from tobacco-related diseases is enormous, but it has faced a number of barriers in this activity. Policy coherence across the federal government first requires a mobilization of the resources and infrastructure within DHHS to reduce current smoking and prevent future smoking.

The CDC, one of 12 DHHS agencies, houses the National Tobacco Control Program under the Office on Smoking and Health (OSH) of the National Center for Chronic Disease Prevention and Health Promotion [18]. CDC-OSH originated in 1965 as the National Clearinghouse on Smoking and Health within the Office of the Secretary of the Department of Health, Education, and Welfare (now DHHS) [19]. CDC-OSH focuses on supporting and coordinating state tobacco control activities with technical assistance for core public health functions. It also develops best practices in tobacco control, conducts surveillance, and raises public awareness on the health effects of tobacco use, primarily but not exclusively through publication of the *U.S. Surgeon General’s Report on the Health Consequences of Smoking*. It also staffs the Interagency Committee on Smoking and Health^2^ (ICSH), formed in 1984 with the mandate to facilitate greater cross-agency collaboration [19].

Under Secretary Tommy Thompson, the ICSH spearheaded the effort that led to the creation of a National Quitline [20]. Reflective of the limited power of the ICSH and DHHS, however, the initial proposal for the Quitline did not receive sufficient funding to fully advertise and staff a strong, nationwide effort to help smokers quit.

Within CDC, centers such as the National Institute for Occupational Safety and Health and National Center for Health Statistics address agency-specific aspects of tobacco control such as worksite exposures and behavioral surveillance, respectively. The CDC’s Division of Maternal and Child Health supports surveillance of maternal and child health (MCH) behavioral issues, targeted prevention programs, and evaluation studies in tobacco control. CDC-OSH, however, faces diminishing appropriations for its core activities. It, as well as other agencies, is also hampered in its mission to achieve comprehensive tobacco control programs at the national and state level by inhibitory government-wide administrative authorities of the Office of Management and Budget (OMB), the White House office responsible for overseeing the execution of the federal budget in Executive Branch agencies.

At the State level, funding for comprehensive state programs is lacking and may be a major reason for the persistence of smoking among more than 45 million Americans [21]. The States are responsible for the public health of their constituents, but the Federal government, through the CDC, can provide leadership, program support, technical assistance, and evaluation to assure the quality and coverage of these activities. In the past, support was provided through NIH research programs (ASSIST) [22], foundations (Robert Wood Johnson Foundation), Block Grants for Chronic Disease Control with program evaluation requirements (CDC), state excise tax-funded programs (such as Proposition 99 programs in California) [23], and settlements of lawsuits under the U.S. Attorneys General Master Settlement Agreement (MSA). Though cigarette excise taxes are collected from smokers and funds from the MSA for the recovery of medical costs from tobacco-related diseases, fewer and fewer of these revenues go to helping smokers quit or to smoking prevention efforts [24,25]. The MSA has been rightfully criticized for not fulfilling its mandate to use substantial resources from the $246 billion settlement for state tobacco control activities. Instead, many of these have been diverted to non-health expenditures.

Within the NIH, the Tobacco Control Research Branch of NCI is the lead agency for behavioral, basic science, and other tobacco control research. In addition to its grant making function, NCI plays a leadership role by partnering with other NIH institutes and non-governmental entities to promote tobacco control research. Although tobacco use accounts for almost 30% of U.S. cancer deaths, tobacco-related research represented only 2.8% of the NCI budget in FY 2003 [26]. The funding imbalance for tobacco reflects a lack of concerted effort to develop budget priorities based on disease burdens within the DHHS more generally [27,28].

The National Institute on Drug Abuse funds and conducts research on a wide range of pharmacological and behavioral studies related to addiction, cessation, and tobacco products. Tobacco is a leading cause of disease in the organ systems under the National Heart, Lung, and Blood Institute funding portfolio. The Fogarty International Center plays a critical role in supporting international tobacco control research. The Tobacco and Nicotine Research Interest Group, a trans-NIH effort to increase collaboration, coordination, and communication on tobacco and nicotine research, is an example of attempts at policy integration within NIH.

Smoking cessation services are provided to specific populations through DHHS agencies. These include Medicare- and Medicaid-eligible populations, veterans, and Native Americans/Alaska Natives (NA/AN), through funding from the Centers for Medicare and Medicaid Services (CMS), Department of Veterans Affairs (VA), and the IHS, respectively, though the coverage of these programs is insufficient. Medicaid – the U.S. public insurance program for the low-income Americans – in particular could provide universally available cessation programs to poor populations at the state level. In 2005, 38 of 50 state Medicaid programs offered at least one form of tobacco-dependence treatment for all Medicaid beneficiaries [29], but these vary considerably in coverage and access. Medicare – the U.S. public insurance program for the elderly – offers only meager support for counseling, and only after a patient has been diagnosed with a tobacco-related disease [30]. The IHS has developed a *Tobacco Control Strategic Plan 2006-08* to improve tobacco use cessation and prevention [31]. Yet over 40% of NA/AN reported current smoking from 1999-2001, nearly 15 percentage points higher than the national average [32]. Thus, tobacco control efforts among NA/AN populations need additional focus to prevent the large tobacco-attributable burden of disease known to affect these populations.

AHRQ currently sponsors the U.S. Preventive Services Task Force, an independent panel of private-sector experts that develops clinical best practices guidelines through extensive reviews of scientific literature [33]. Similarly, the Task Force on Community Preventive Services, a non-federal group appointed by the Director of the CDC, reviews the scientific literature to develop best practices guidelines for use in community-based programs to prevent tobacco initiation, increase cessation, and reduce exposure to second hand smoke [34]. In 2008, the Public Health Service-sponsored Clinical Practice Guidelines to treat tobacco used and dependence was released asserting ten key recommendations supporting smoking cessation [35].

The Substance Abuse and Mental Health Services Administration (SAMHSA) has a mandate, through what is known as the Synar Amendment^3^, to enforce a state minimum tobacco product purchase age, conditioning state substance abuse block grants on compliance; in FY 2006, all states were reported to be in compliance with establishment of minimum age laws, but enforcement is still problematic [36].

Other DHHS Agencies have a variety of concerns and jurisdictions relative to tobacco control. These include international negotiations under the Office of Global Health Affairs, minority health concerns under the Office of Minority Health, and the publication of Surgeon General Reports on smoking and health through the Office of the Surgeon General (Figure 1).

### Non-health Agency Tobacco-related Concerns

The Federal Trade Commission (FTC), a consumer protection and fair competition agency within the USG, is responsible for ensuring that information on cigarette packages are accurate and for monitoring the accuracy of claims made in tobacco advertising. Because its enforcement efforts are within a trade framework, it has generally been tolerant of claims made in advertising [37]. Under the Federal Cigarette Labeling and Advertising Act of 1965 [38], the FTC was given authority over cigarette package warning labels. The FTC oversees the rotation of the four current Surgeon General’s warnings for cigarettes and smokeless tobacco. It also collects data on the marketing expenditures of tobacco companies and conducts tests to assess the tar and nicotine levels of cigarettes.

There is general agreement that the machine-based method the FTC uses to measure tar and nicotine yields from smoking, called the Cambridge Filter Method, is faulty. A smoker’s exposure to tar and nicotine tend to be higher than measured through the Cambridge method because smokers take larger, deeper, or more frequent puffs on cigarettes that measure lower tar and nicotine yields in order to obtain the nicotine dosage they need. As a result of these testing flaws, the FTC (supported by a judicial ruling in Federal courts) has recently asserted that advertisers should not include phrases that state or imply FTC endorsement of tar and nicotine yield data produced from by the Cambridge method [39].

With the creation of the Department of Homeland Security, responsibility for the collection and enforcement of tobacco excise taxes has been split between the Departments of The Treasury and Justice. The Treasury Department, through the Alcohol and Tobacco Tax and Trade Bureau, is responsible for the collection of tobacco excise taxes. The Bureau of Alcohol, Tobacco, Firearms and Explosives (ATF) within the Department of Justice (DOJ) is responsible for monitoring and eliminating smuggling of tobacco across state borders and from other countries into the United States. Rates of smuggling are unknown, but it is considered a significant loss of revenue for the federal government and a potential source of criminal activity and funding source for terrorists. For theses reasons, ATF officials and other agencies have stepped up their efforts to reduce smuggling [40]. From the perspective of tobacco control, smuggling undermines price controls that act as a deterrent to smoking, especially among youth.

The Environmental Protection Agency (EPA) conducts research and disseminates information on indoor air quality as part of its broader responsibilities. In 1992, it issued an important report, *Respiratory Health Effects of Passive Smoking: Lung Cancer and Other Disorders*, which concluded that second hand smoke is a Group A, human lung carcinogen and that it has significant deleterious health effects on children [41]. Its regulatory authority to enforce clean indoor air rules has been undercut, however, and should be revisited in the new Administration. The EPA has also begun working with DHHS on a nationwide outreach campaign to families of low-income children on the health effects second hand smoke, especially asthma in children.

U.S. Department of Agriculture (USDA) has been concerned mainly with reporting tobacco trade, production, exports, imports, and international patterns as an agricultural and economic concern, not a health concern. Data from USDA have been important to understand and monitor patterns of consumption as well as smuggling, taxation effects, and tobacco trade issues. The USDA, however, has discontinued tobacco as a research area within its Economic Research Service. October 2007 was the last publication of *Tobacco Outlook*, a USDA publication of research in tobacco production, consumption, prices, stocks, imports and exports, and consumption and trade of tobacco products [42]. USDA no longer conducts research in any of these crucial areas, leaving the industry-supported *Tax Burden on Tobacco*, prepared by tobacco industry consulting firm Orzechowski & Walker, the only source of similar information. The USDA’s termination of research on tobacco will leave no mechanism to evaluate the effects the recent 62 cent increase in federal excise tax on tobacco products.

The U.S. Department of State (DOS), along with the U.S. Commercial Service, is barred from spending funds to promote the sale or export of tobacco products or to ease foreign country restrictions on tobacco or tobacco product marketing via the Doggett Amendment. A similar prohibition is in place for the international arm of the Department of Agriculture through what is known as the Durbin Amendment [43].

The U.S. Trade Representative (USTR) has defended the opening of markets for and fair trade of U.S.-produced tobacco products abroad [44]. This is guided by international agreements for participation in the World Trade Organization (WTO), specifically, the Generalized Agreement on Tariffs and Trade (GATT). In the Clinton Administration, there was some attempt to incorporate health concerns into potential actions taken by USTR to defend U.S. tobacco exports abroad [45], but was not carried forward through the Bush Administration.

# Tobacco control priorities at the Federal level

Three unresolved tobacco control policy issues require particular attention within the federal government: ratification of the World Health Organization (WHO) Framework Convention on Tobacco Control (FCTC), authorizing the FDA to regulate tobacco products, and settling the U.S. Department of Justice’s Racketeer Influenced and Corrupt Organizations Act (RICO) case against the tobacco industry. Each of these three issues are discussed in the next section as part of a policy coherent plan for the federal government on tobacco control, but highlighted here for their significance and potential impact.

The FCTC is the first-ever global public health treaty [46]. It was developed to counter the globalization of tobacco and the growing burden of disease from tobacco use worldwide. The treaty is a comprehensive agreement that addresses both supply and demand components of tobacco use with 161 countries that have signed on as party to the treaty. Though it was signed by then-DHHS Secretary Tommy Thompson, President Bush never sent it to the Senate for ratification. Ratification of the FCTC should be a leading priority within health reform for the Obama Administration. It will provide the needed impetus to revive comprehensive tobacco control efforts at the national level and act as a framework for developing a national tobacco control agenda. The articles of the FCTC address areas that make up a comprehensive national plan as well as providing guidelines for implementation. Ratification of the FCTC will also help the United States repair its international reputation, and provide international accountability to the U.S.’s tobacco control efforts.

The current bill to grant FDA regulatory authority over tobacco products has been a work in progress for over five years [4]. Until 2008, it had never passed in either house of the U.S. Congress, but it was voted through the House of Representative in 2008 and again in April 2009. There is little dispute over whether the FDA should regulate tobacco products; there is controversy, however, over whether the existing bill is the appropriate legislation to grant that authority. Despite strong support for the bill by public health groups, critics argue that Philip Morris should have no role in drafting the legislation, industry representatives should not be included as non-voting members on the oversight committee, the FDA should not have limits on its authority to ban classes of tobacco products, and the bill may preempt more active legislation at the State level. In addition, it makes an explicit exemption on flavoring restriction for menthol cigarettes that are smoked mainly by African Americans and thus may facilitate smoking among this population. Granting FDA authority over tobacco products, along with ratification of the FCTC, should be a high priority within health reform. It is important, however, that the Obama Administration work to ensure that the legislation supports the strongest possible language and conditions beneficial to public health without concessions to the tobacco industry.

Federal Judge Gladys Kessler found the tobacco industry had violated two sections of the U.S. Racketeer Influenced and Corrupt Organizations Act (RICO) in the U.S. Department of Justice’s case against the tobacco industry, *United States v. Philip Morris* [47]. Judge Kessler asserted in her decision that the tobacco industry had engaged in behavior to defraud the American public for over 50 years. Though the case is currently on appeal, Judge Kessler’s Remedies for corrective action should be pursued vigorously. The case is an opportunity for the USG to order the tobacco industry to cease false and deceptive activities, obtain some regulatory authority over tobacco products, and assert greater oversight of the industry through disclosure of industry documents and disaggregated market data.

Bolstering the smoking cessation capacity of the health care system will also be a tobacco control priority at the federal level if the Obama Administration successfully advances its health care reform agenda. A commitment to prevention, long neglected within the U.S. health system, is an essential component of health care reform in the United States [1,48]. Successful reform of the U.S. health care system into a universally accessible, prevention-oriented system would include a strengthened infrastructure to support smoking cessation. A prevention-oriented health system would add smoking as a vital sign on medical records, improve health care provider training and clinical reminder systems for tobacco use cessation, provide insurance coverage for cessation services and products, integrate clinical with behavioral treatments, and expand the capacity of state and national quit-line services [49].

## **Expanding government tobacco control efforts**

The capacities of DHHS and other federal agencies have not been fully engaged in a unified and coherent tobacco control effort at the national level. The current financial and political environment makes it difficult for the USG to contemplate any new programs aside from reviving the economy and seeking a resolution to the conflicts in Iraq and Afghanistan. Tobacco control is nonetheless a critical element of the domestic health care reform agenda for two reasons. First, it can be initiated at little to no cost to the government. Creating smoke-free environments, engaging in public education, and focusing research and surveillance on tobacco control are simple first steps that require little more than asserting tobacco control as a national public health priority. Programs and policies that require government expenditure, including a national media campaign and provision of smoking cessation services, can be introduced as the country’s economic condition stabilizes. Second, the economic return on investment in tobacco control could be quite remarkable. A $1.8 billion investment in California’s state tobacco control program, for example, yielded a savings of $86 billion in personal health care costs alone [50], mirroring potential estimated cost savings of 5.6% for state funded medical programs if states were to implement comprehensive smoking prevention and cessation programs [51]. Thus, renewed commitment and mobilization within the programs and agencies described above may be critical components of any health reform strategy for the new Administration.

### Guiding Principles for a National Tobacco Control Agenda

The National Tobacco Control Agenda effort should be

- spearheaded by the clear, unambiguous leadership of the Administration, supported by legislation, and coordinated throughout the Federal government;
- cognizant of the health and economic burden of tobacco use in the United States and internationally;
- a collaborative effort coordinated across agencies;
- global in scope with a commitment to global health that is supportive of low- and middle-income countries in their efforts to reduce the future impacts of tobacco use.

Specific Recommendations

Even without ratification of the FCTC, the USG should move forward toward implementing a comprehensive National Tobacco Control Agenda. The FCTC provides a useful blueprint for comprehensive national action for many countries with more centralized health programs, and it can be applied to the federal system of government in the United States. Below we outline specific actions the USG can take in order to implement a National Tobacco Control Agenda based on the provisions of the FCTC.

Tax and price measures. Raising excise taxes on tobacco products, like regulation of packaging and labeling of tobacco products and banning of advertising, promotion, and sponsorship, requires Congressional action before policy changes can be made. Recently, Congress approved a 62-cent excise tax increase on tobacco products to finance the State Children’s Health Insurance Program (SCHIP). In addition to the financial base the increase tax provides to the SCHIP program, increasing taxes is an effective price measure for reducing consumption of tobacco products [15]. At the state level, tobacco control programs funded by increases in state excise taxes have shown the public health benefits that can be made when taxes are invested into tobacco control programs [52,53]. Congress, with Presidential leadership, should enact additional increases in excise taxes on tobacco products as a means of reducing consumption as well as funding federal tobacco control programs.

Other prices measures besides taxes should also be considered to reduce the consumption of tobacco products. Increased production costs due to payments from the tobacco industry to states as part of the MSA, for example, have been passed on to consumers as price increases. Shifting costs for FDA regulation of tobacco products to the tobacco industry and requiring monetary penalties related to the tobacco industry violation of the RICO Act are two pricing measures the USG should pursue to reduce tobacco consumption and strengthen tobacco control efforts.

*Protection from exposure to second hand smoke*. An estimated 34.8% of Americans are covered by 100% smoke-free workplace, restaurant, and bar laws [54], but the federal government can help to further reduce exposure of Americans to second hand smoke (SHS). The USG can model the importance of smoke-free environments by adopting more widely the smoke-free meetings policies of CDC, NIH, and other agencies and smoke-free campus policies adopted by CDC and NIH. The Occupational Safety and Health Administration^4^ (OSHA) in particular can take a range of actions related to worksites to assure 100% smoke-free environments, consistent with EPA concerns for clean indoor air [41]. Any OSHA regulation, however, must be considered with respect to the unintended weakening of state and municipal workplace smoke-free laws through federal pre-emption. Any USG efforts should establish the floor, and not the ceiling, of worksite tobacco control efforts at the state level.

Only 38% of schools, 55.4% of school districts, and 63.6% of schools in the United States prohibit all tobacco use in all locations [55].The Department of Education must act swiftly to enforce of Public Law 103-227. Passed into law as the Pro-Children Act of 1994, Public Law 103-227 prohibits “smoking within any indoor facility owned or leased or contracted for and utilized by such person for provision of routine or regular kindergarten, elementary, or secondary education or library services to children” [56]. The law mandates that any program receiving federal funds must ban indoor smoking, including schools, day care programs, libraries, and WIC and Head Start centers.

The Department of Housing and Urban Development can provide public education on the dangers of SHS in the home, spearhead demonstration projects to assure smoke-free homes (including multiple unit dwellings that may circulate SHS), and integrate clean indoor air policies in its public housing and community development projects.

The Department of the Interior has an opportunity to advance outdoor smoke-free policies in the lands under its jurisdiction, especially national parks. Smoke-free policies, which currently vary across parks, can protect visitors, reduce wildfire risks, and prevent litter.

The DOD issued its smoke free workplace policy through Directive 1010.15 [57] and later Instruction 1010.15 [58] which created smoking prohibitions throughout the armed services. Many exceptions, however, were granted, including ships, indoor smoking areas, housing, and Morale, Welfare, and Recreation facilities. Because of the importance of the health of military servicemen and women, smoke-free bases, ships, and facilities and the elimination of tobacco sales from commissaries and exchanges should be required; collusion between the DOD and tobacco industry to maintain a culture of smoking in the military must be stopped [59].

Similarly, tobacco product sales should be eliminated from all USG occupied facilities, whether owned by the USG or through contract arrangements. This could be accomplished by Executive Order.

*Tobacco products contents and disclosures.* FDA authority over tobacco products is an idea that has been circulating within the federal government for at least two decades and received the most attention in 1996 when the FDA, then led by David Kessler, asserted regulatory jurisdiction over tobacco products as drug delivery devices [60]. The proposed regulation was struck down by the Supreme Court in 2000 [61]. Granting FDA regulatory authority over tobacco products is an essential part of a national tobacco program. Strong legislation will require, among other things, tobacco companies to disclose chemical compounds found in tobacco products and tobacco smoke, grant FDA authority to promulgate regulations on cigarette testing and product standards, develop standards for evaluating novel products, ban misleading terms that may imply reduced risk, and update and revise warning labels to promote greater public understanding of the risks of using tobacco products [16]. The current version of FDA regulation of tobacco is subject to substantial criticism from several perspectives (see above).

*Packaging and labeling of tobacco products.* Oversight of tobacco product packaging and labeling is granted by Congress to FTC. The Federal Trade Commission Act [62] grants the FTC authority over deceptive packaging tobacco products as a measure to ensure fair trade. The Federal Cigarette Labeling and Advertising Act of 1965 [38] places the FTC in charge of overseeing rotating warning labels on cigarettes and smokeless tobacco. Any change in regulatory authority over packaging and labeling must come from legislative mandate granted by Congress. The importance of a strong bill to grant FDA authority over tobacco products is thus essential, given the level of influence the tobacco industry has in Congress and its ability to influence single pieces of legislation. Granting FDA authority over tobacco products can act as a means regulating the packaging and labeling of tobacco products without obtaining Congressional approval.

Absent FDA regulatory authority, state consumer protection laws may be a new avenue to change tobacco product packaging. The U.S. Supreme Court recently decided that tobacco companies can be sued by consumers under state consumer protection laws for using misleading terms such as “light” to imply healthier cigarettes. If lawsuits are successful, tobacco product packaging may not only change with respect to use of misleading descriptors but in other ways consistent with the implementation guidelines for the FCTC.

*Education, communication, training, and public awareness.* Raising public awareness of the dangers of smoking and the strategies of the tobacco industry are a crucial component of a national tobacco control agenda. Tobacco control public education, technical assistance, and state support functions of CDC are limited by the funding for OSH. As the lead agency on tobacco control, funding for CDC-OSH must be increased to allow it to expand and strengthen its work. The USG, through collaboration among CDC, NIH, and non-governmental organizations such as the American Legacy Foundation, should fund and increase support for a national mass media counter-marketing campaign as a public education effort to raise awareness and de-normalize tobacco use [63].

The lack of attention to effective school-based health programs that address smoking has allowed the tobacco industry to infiltrate schools with tobacco education programs that have proved to be counterproductive [64]. The tobacco industry should not be allowed in schools or support partners in the development of any school-based tobacco control measures, regardless of their stated intentions. School boards should follow IOM recommendations for smoking prevention programs [16].

The USDA, through its administration of WIC (a food program for low-income pregnant women and women with children under age five) and support for 4-H clubs (a youth development program), can also undertake a youth-oriented educational campaign that reaches young mothers and rural populations in particular.

*Advertising, promotion, and sponsorship*. Bans on tobacco advertising are limited. The Public Health Cigarette Smoking Act of 1969 banned cigarette advertising on television and radio and the Comprehensive Smokeless Tobacco Health Education Act of 1986 banned smokeless tobacco advertising on television and radio. Federal legislation banning television and radio advertising also preempts states from banning cigarette advertising or promotion for health-related reasons. Preemption of state and local bans on tobacco advertising and promotion should be removed, allowing states to regulate tobacco industry marketing for health related reasons.

The MSA includes limits on tobacco industry sponsorship or events. It prohibits brand name sponsorship of concerts; football, basketball, baseball, soccer, and hockey leagues, teams or games; events whose intended audience has a significant percentage of youth; and events where any paid participants are youth. Tobacco companies are allowed to sponsor one event by brand name in any 12-month period provided it is not a prohibited brand name sponsorship with some restrictions. Tobacco companies are allowed to continue sponsorships of events by corporate name and events in adult-only facilities, such as bars, by brand and corporate name.

As with packaging and labeling of tobacco products, FDA regulatory authority over tobacco products is an essential action to fill in existing gaps in restricting brand and corporate advertising, promotion, and sponsorship.

The impact of smoking in movies on youth smoking requires the inclusion of the entertainment industry as a stakeholder in a national tobacco control agenda [63].

*Smoking Cessation*. A subcommittee of the ICSH has made six recommendations for federal action to promote smoking cessation: establishing a National Quitline; launching a media campaign to encourage smoking cessation; providing insurance coverage for tobacco dependence treatment for individuals covered under federal health plans including Medicare and Medicaid, federal employees, and DOD personnel, among others; creating a new tobacco research infrastructure; reforming the clinical training infrastructure to ensure clinicians are adequately trained and have the resources and knowledge needed to intervene with patients who use tobacco; and initiating a Smoker’s Health Fund to provide a financial base to help smokers quit [20]. Of the recommendations submitted to then-Secretary of Health and Human Services Tommy Thompson, only one – the establishment of the National Quitline – has been implemented. The National Quitline, however, has not been well publicized, nor does it currently have the capacity to handle a high call volume that would result from a large media campaign promoting smoking cessation. In order to make smoking cessation services universally available and effective, greater steps toward implementing these recommendations must be taken.

For instance, Executive Order 13058 encouraged the heads of USG agencies to establish smoking cessation programs. Support for smoking cessation programs for federal employees should be strengthened and, as with all smoking cessation programs offered or covered by federal programs, including those offered to the federal prison population, should adhere to best practices. Currently, the Federal Employees Health Benefits program does not mandate that its health insurance providers cover smoking cessation services that follow Public Health Service guidelines. As a model being considered for extending universal health coverage to all Americans, the Federal Employees Health Benefits Plan should mandate smoking cessation services be covered in all health insurance plans, including any plan being developed to provide universal health care.

The Joint Commission, a health care organizations accrediting body, should strengthen its current requirements for smoking cessation beyond mere provision of written information to provide incentives for health care organizations to provide smoking cessation services. Federally-funded and federally authorized health programs should provide comprehensive coverage for smoking cessation services [65]. Monitoring and evaluation of these programs to demonstrate success and high level support should be expanded, as the benefits of smoking cessation to the health of Federal employees (including reduction in health care costs, absenteeism, and exposure to SHS) is quite clear [8]. All agencies that provide health services to non-federal employees should adopt similar approaches.

Of particular importance is the provision of smoking cessation services and products through the VA. The smoking rate among soldiers in and returning from Iraq is more than twice the rate of the general population (52% vs. 19.8%) [66,67], making smoking cessation services, and averting premature deaths among veterans, an urgent need in the VA. Any barriers to receiving smoking cessation services through the VA’s National Smoking and Tobacco Use Cessation Program must be eliminated.

*Illicit trade in tobacco products.* The problems related to smuggling highlight the poorly managed efforts of the USG to monitor and track tobacco products. Excise tax collection, smuggling of cigarettes, counterfeit foreign cigarettes, and tracking of production, consumption, and trade of cigarettes are interrelated monitoring activities that are fragmented and spread across the Treasury Department, DOJ, and formerly the USDA. This fragmentation should be consolidated and, in the case of USDA research activities in tobacco, revived.

*Sales to and by minors*. In addition to strengthening state enforcement of youth access restrictions mandated by the Synar Amendment, the USG must improve monitoring of Internet tobacco product sales to ensure minors are not accessing tobacco. Internet sales can be banned altogether through the interstate commerce power of Congress. They can also be regulated by the Treasury Department to ensure collection of tobacco taxes.

*Alternative activities*. Since the 1930s, the USG has had a federal price support program for tobacco guaranteeing tobacco prices and limiting tobacco marketing [68]. In 2004, the Fair and Equitable Tobacco Reform Act ended a government sponsored quota and subsidies program for tobacco farmers. In lieu of prices supports, the USG created the Tobacco Transition Payment Program to assist tobacco quota holders and producers to transition to the free market for tobacco [69] and offset any loss in diminished value for their farms and the loss of future support. The program, which would disperse $9.6 billion over ten years to tobacco quota holders, is funded by an assessment on tobacco manufacturers and exporters [70].

Financial support alone, however, are not sufficient for transitioning farmers out of tobacco production. There is no specific provision within the Tobacco Transition Payment Program to assist former tobacco quota holders to transition to other agricultural activity. There are numerous services, however, offered by the USDA to support the agricultural sector that can be accessed by former tobacco farmers. The USDA must actively work with tobacco-dependent communities to take full advantage of existing economic development resources that allow them to successfully transition out of tobacco farming. The USG should create incentives and offer technical assistance for tobacco farmers to voluntarily transition to the production of other crops, initiate non-agricultural business ventures, or engage in other programs such as occupational retraining to facilitate the economic diversification of tobacco-dependent communities.

*Environment and health of persons.* Tobacco is a pesticide-intense crop, and the effects of pesticides, as well as other agricultural practices, in tobacco growing require greater attention. Though risks associated with pesticide residue on tobacco have been found to be negligible for smokers, the risk to tobacco agricultural workers may be significant [71] as may the environmental impacts. The USDA should revive its monitoring of agricultural practices associated with tobacco growing and ensure that plants used in tobacco products do no additional harm to workers, smokers, or the environment.

*Liability*. In September 1999, the DOJ filed a case against the tobacco industry to hold it accountable for illegal and harmful practices under the RICO Act. The presiding judge, Gladys Kessler, found that the tobacco industry had engaged in behavior to defraud the American public and violated two sections of the RICO Act. Her Final Judgment and Remedies called for companies to cease racketeering activities and deceptive practices; make public corrective statements on the adverse health effects of smoking, addictiveness of smoking and nicotine, and lack of health benefit from cigarettes labeled “light,” “mild,” or other similar descriptors; cease manipulation of cigarette design and composition to deliver optimum nicotine delivery; and publicly disclose industry documents and disaggregated market data. The ruling, currently on appeal, is an opportunity for the federal government to aggressively pursue tobacco control. Corrective actions from the decision should be sought through remedies that will have a strong public health impact.

Litigation as a tobacco control strategy in general has been variably successful in the United States and abroad [15,72]. It has forced tobacco companies to face tobacco control advocates in an arena where a neutral and powerful third party has the ability to enact penalties on tobacco industry malfeasance. The federal government should place no obstacles in the way of litigation against tobacco companies or provide any protections for the tobacco industry, allowing the judicial process to play out in both individual cases and class action cases.

*Research and Surveillance*. In order to better understand the vulnerabilities related to tobacco use, co-morbidities of tobacco use, the epidemiology of tobacco-related disease, the nature of nicotine addiction, effectiveness of cessation and prevention strategies, and implementation of practice-based research, a much higher commitment to funding of tobacco-related research must be made. A mechanism similar to the Tobacco Research Implementation Group, which made recommendations to increase NCI’s tobacco funding portfolio [73], should be created and given the political and fiscal support needed to identify existing gaps in research and to bolster the tobacco-related research agenda.

Similarly, surveillance of tobacco use behaviors and tobacco-related diseases need continued support and expansion. Numerous surveillance tools exist, including the National Health Interview Survey, Behavioral Risk Factor Surveillance System, Current Population Survey Tobacco Use Supplement, National Survey on Drug Use and Health, Adult Tobacco Survey, Youth Tobacco Survey, and Youth Risk Behavior Surveillance System [74]. There are gaps, however, in the topics covered by existing surveys, and each has methodological limitations. Information on brands of cigarette smoked and use of menthol and light cigarettes as well as “potential reduced-exposure products” is scant as is information on use of smokeless tobacco and cigars [74]. Methodological challenges include improving the rate at which surveillance systems can collect, analyze, and disseminate data; broadening surveys to capture special populations including ethnic minorities, gay, bisexual, lesbian or transgender communities, homeless and prison populations, the mentally ill, and individuals with disabilities; increasing survey response rates; and collecting longitudinal data [74].

One particularly challenging area of surveillance is monitoring the activities of the tobacco industry. The pro-tobacco business strategies of the industry are important to monitor as a determinant of tobacco use. Current data sources to monitor tobacco industry activities – including tobacco product pricing, smuggling of cigarettes, lobbying and campaign contributions, corporate donations, public relations, advertising, and multiple forms of product marketing – are highly fragmented, uncoordinated, and incomplete [75]. Recommendations to create a national research initiative on tobacco marketing activities and develop a clearinghouse for tobacco industry activity should be implemented, as well as longer term commitments to develop a surveillance system to monitor the tobacco industry [75].

In order to achieve the surveillance needs in tobacco control, oversight authority given to OMB to approve federal surveys and to manage scientific information through the Data Quality Act of 2001 must be relaxed or removed. The Data Quality Act allows individuals and companies to challenge government information they consider inaccurate, and raises the standard of scientific certainty on which regulations are based to an almost unreachable level. The Act has been a boon to corporate interests that can use it to challenge government regulations [76] and allows OMB greater control to expedite or delay the release of scientific information.

## *International Cooperation and U.S. Foreign Policy on Tobacco and Health*

*Trade policy*. The Global Burden of Disease project points out the enormous impact of non-communicable disease on the health of low- and middle-income countries as well as on emerging and established economies [77]. It is in the interest of the USG to support effective global health collaborative actions on tobacco use, which will be responsible for 10 million deaths and enormous economic development challenges in the coming decades [78]. Thus, sensible trade policies that recognize health as a key foreign policy objective are needed.

One function of the DOS is to monitor and collect data on foreign markets and facilitate U.S. business interests overseas. The interests of the tobacco industry were given equal consideration by the DOS until the 1990s, when first the USDA Foreign Agricultural Service (through the Durbin Amendment in the Agricultural Appropriations Act for FY 1993) and later the Department of Commerce, DOJ, and DOS, under an amendment to the FY 1998 Commerce, Justice, and State Appropriations Act put forth by Representative Lloyd Doggett, were prohibited from spending funds promoting the interests of the tobacco industry overseas (except in cases of discriminatory policies) [79].

Enforcement of both the Durbin and Doggett Amendment has been lax under the Bush Administration. U.S. House of Representatives member Henry Waxman and U.S. Senator Richard Durbin have noted violations of both statutory prohibitions. The U.S. Commercial Service provided information to United States Tobacco International Incorporated (a wholly owned subsidiary of United States Tobacco) on the smokeless tobacco market in Pakistan, and the U.S. Foreign Agricultural Service worked on tobacco growing in China, Greece, Pakistan, and Moldova [43]. Also, Foreign Agricultural Service officials have assisted the U.S. Trade Representative during trade negotiations on tobacco, which may be construed as a violation of the Durbin Amendment.

The regulatory authority of the Durbin and Doggett Amendments is tenuous because they are additions to appropriations bills that must be renewed annually. Also, the specific guidelines and regulations issued annually are subject to the policies of the Administration in office. The provisions of the Durbin and Doggett Amendments should be made into law. Strong and clear guidelines to eliminate any support for expansion of tobacco markets should be communicated to overseas staff and vigorously enforced.

Tobacco products should be treated as exceptional goods with respect to health in trade negotiations. That is, instead of being considered a normal good subject to trade agreements, tobacco and tobacco products should be exempted from stipulations of trade agreements as a necessary action to protect public health. The manufacture and sale of tobacco products across national boundaries are subject to WTO agreements, including the GATT, Technical Barriers to Trade (TBT), Agreement on Agriculture, and the General Agreement on Trade in Services (GATS) [80]. Tobacco and tobacco products should be removed from the stipulations of multi- and bi-lateral trade agreements, allowing other countries to strengthen national tobacco control measures and import restrictions without having to fear retribution from trading partners. As a precedent, the U.S-Vietnam Free Trade Agreement excludes tobacco from its tariff reduction plan [81]. At a minimum, any requirement to show how free trade in tobacco will cause harm should be reversed; tobacco is simply not a normally traded good and thus should not be protected by trade policy. Tariffs on tobacco and tobacco products entering the United States can be increased, providing opportunity for trade partners to raise their own tariffs on tobacco products. In the short run, the USTR should be instructed by Executive Order to consult DHHS on any actions requested by U.S. tobacco manufacturers prior to invoking trade sanctions on countries accused of unfair trade practices regarding U.S. tobacco products.

*FCTC*. Negotiations on the WHO FCTC were supported by the White House Domestic Policy Council (DPC) and led by the DHHS Office of International and Refugee Health (now Office of Global Health Affairs) during the late 1990’s. The treaty is a comprehensive international agreement covering measures to reduce the demand of tobacco (i.e., price measures, protection from second hand smoke, regulation of tobacco product content and product disclosures, packing and labeling of tobacco products, public education, tobacco advertising and marketing, and smoking cessation) and the supply of tobacco (i.e., illicit trade in tobacco products, sales to and by minors, provision of support for economically viable alternative activities) [46].

The U.S. Secretary of DHHS under the Bush Administration signed the treaty, but the FCTC has not been submitted to the Senate for ratification. The FCTC is the first global public health treaty and, even without ratification, can be used as a template for the USG to focus its national tobacco control efforts. The United States can reestablish a position of international leadership on tobacco control by ratifying the treaty and moving quickly to begin its implementation through actions at the state level. In the U.S. federal system, these actions are necessary to assure compliance with international agreements; there are precedents for this found in international environmental agreements such as the Vienna Convention and Montreal Protocol [82].

Concerns have been raised about state compliance with FCTC binding obligations and over violation of constitutional rights to free speech by restrictions on tobacco advertising. However, the FCTC makes allowances to accommodate differences in national governmental structure and laws. Upon ratification, the United States will be subject to the requirements of the treaty but able to tailor its implementation to the unique circumstances presented by the U.S. governmental and legal context.

The United States can also provide foreign aid and technical assistance to support other countries in implementation of the treaty. These efforts may involve NIH to support international research, CDC to support program implementation, and USDA to support crop diversification.

The U.S. Agency for International Development (USAID) has a history of promoting tobacco growing as a development strategy, but adopted a policy in 1999 to cease the support of tobacco as a cash crop or the support of agribusiness activities contributing to tobacco production, promotion, or use [83]. Beginning in 2006, USAID made strengthening the linkage between global tobacco control and its performance goals official policy [84]. It has never had a formal program, however, to provide foreign aid in tobacco control, either in its health programs or economic development activities. U.S. development assistance has recently been brought under more direct control of the DOS, but with PEPFAR occupying the central role in health-related foreign aid, little attention is being paid to non-communicable diseases such as those related to tobacco use.

Tobacco control can, and should, be incorporated into USAID health programs. For example, aid could be provided to include smoking cessation as part of tuberculosis control programs^5^, smoking prevention in MCH programs to reduce adverse reproductive outcomes (such as abortion, low birth weight, and infant mortality), and support for tobacco control in other health system development programs. Opportunities for cross-agency partnerships between the DOS, CDC and NIH to develop the tobacco control expertise and technical assistance capacity of international partners and even within the U.S. foreign service could be expanded.

**Summary and conclusions**

The wide public support and increasing political momentum for health care reform is an opportunity to advance the progress of tobacco control efforts, reducing the health toll on Americans and the economic burden on the American health care system. National plans to substantially reduce the burden of tobacco use have been put forth [16,17] and the FCTC provides a framework for developing a comprehensive national tobacco control plan. Yet those plans and framework are unlikely to be fully realized without the strong and committed leadership of the Office of the President and support of the Congress. In light of existing policies and programs and opportunities for expansion of tobacco control within the federal government, the guiding principles above lead to the following recommendations for action.

# *Presidential Leadership*

President Obama should make a strong public commitment and begin to mobilize the vast capacity of the USG to achieve policy coherence in tobacco control. The President should designate an Executive Office-level lead to develop a national tobacco control strategic policy. Coordination and leadership for a national tobacco control plan should be centralized in a highly visible and reputable lead agency such as DHHS or the White House Office on Health Reform with additional programmatic resources drawn from other agencies (for example, CDC, FDA, NIH, SAMHSA, and IHS in DHHS, and as an interagency effort, USDA, USTR, and the other Cabinet-level departments). Lead agency staff would coordinate the programmatic activities with support from the White House in a manner similar to the DPC’s coordination of the U.S. FCTC negotiations under President Clinton. White House involvement will assure a comprehensive and integrated national tobacco control program across all USG agencies. Financial commitment from the U.S. Congress and political support from the White House would facilitate participation from these agencies. The ICSH may provide a model for a convening function that is elevated to the level of the DPC.

Agency reorganization to facilitate interagency cooperation is a particularly challenging aspect of achieving policy coherence for a national tobacco control agenda. For example, the ICSH is a forum designed through Congressional mandate to facilitate interagency collaboration in tobacco control that has failed to yield significant policy or programmatic changes. Its work on a national tobacco cessation plan has been its most successful effort in recent year, but resulted in only the underfunded National Quitline. Yet it is possible to foster interagency collaboration in tobacco control is the appropriate leadership and political emphasis is provided. Early USG activity on the FCTC brought together representatives from DHHS, DOS, DOC, FDA, DOJ, and the Treasury Department together to develop the positions that would satisfy the treaty but accommodate agency concerns. This process mobilized multiple agencies because it was given prioritized by the Clinton Administration and led by the DPC, a politically important White House administrative body. Similarly, strong presidential commitment and leadership to tobacco control as part of the Obama Administration’s health care reform efforts would be necessary to mobilize government agencies for interagency collaboration and policy coherence.

Further, combined action by the White House and the U.S. Congress is needed to create and implement an effective national tobacco control strategy. The Obama Administration must provide leadership through the budget request to Congress. Concomitantly, the President must work with members of Congress for sponsorship of the appropriate authorizing legislation to secure long-term support. A strong and well-supported communications effort would be needed to assure public support. Over time, a White House Office on National Tobacco Control Policy or tobacco control division within the White House Office of Health Reform could be created similar to those for AIDS and drug control.

# *Participation and Buy-In*

A comprehensive national tobacco control plan would require broad-based participation and buy-in beyond the government agencies typically thought of as responsible for tobacco control. The lead government agency should work with partners such as the American Legacy Foundation, American Cancer Society, American Lung Association, American Heart Association, Campaign for Tobacco-Free Kids, Partnership for Prevention, American Medical Association, American Public Health Association, and other professional organizations and societies to develop public support for the program. A broadly inclusive and neutrally facilitated process of stakeholder consensus-building could be used to develop the central policies of a national tobacco control plan, with specific details worked out by sub-groups of active participants. Ideally, this forum would report back to Congress on fiscal matters and to the White House on programmatic matters. The process used to determine Healthy People goals and strategies may be a model for achieving this level of participation.

## *Interaction with the States*

Most successes in tobacco control programming have been at the state and municipal level. Much of the progress in state and local tobacco control programs has been made with the assistance and leadership from the CDC-OSH National Tobacco Control Program. The CDC not only provides states and municipalities with technical assistance to bolster their programs, but has demonstrated leadership in providing a framework to weave them into an interconnected network of programs [15].

In contrast to the positive interaction with CDC-OSH and the National Tobacco Control Program, state and local governments are wary of federal legislative action on tobacco control, and often rightly so. Concerns over preemption of state and local legislation by weak federal laws are bolstered by the use of preemption as an explicit tobacco industry strategy to subvert local tobacco control efforts. Additionally, the federalist structure of the U.S. government has left most, if not all, responsibility for public health in the hands of states and local government. This limits the authority of the federal government to enact tobacco control measures without impinging on the authority of states and local government to safeguard the health of their residents.

Federal standards or legislation that overlap with the ability of states to protect public health should always be considered a policy floor that state and local government can build on. For example, preemption over state actions on many retail aspects of advertising and promotion is a part of the proposed FDA bill. The actions of the federal government, appropriately adjusted to address concerns over preemption, should act to complement and bolster those of the states.

Even with success in passing tobacco control laws at the state and local level, federal involvement in tobacco control is needed for four reasons. First, there are some actions that are beyond the ability of states. For example, some activities, such as product regulation through the FDA and controlling interstate and international smuggling, are of outside of state capacities. Second, the infrastructure of the federal government is so vast as to enable a coordinated and directed tobacco control program across states. There are also expertise and resources for tobacco control available in the federal government that outweigh those available in states. Third, federal involvement can help to strengthen the tobacco control efforts of states that lag behind other states with more successful programs. Finally, some areas, such as foreign policy and the military, fall solely within the purview of the federal government.

There are numerous tools at the disposal of the federal government to affect policy change at the State level, including administrative rulemaking (see for example the National Minimum Drinking Age Act of 1984 [85]) and issuance of standards and guidelines. In other of areas, the federal government has sole authority for policy making. Foreign policy, including trade, diplomacy, and development aid, must be aligned with domestic policy to reduce tobacco use globally.

Executive orders (EO) are a tool that a president can use to make an immediate impact on policy, though adherence to them varies. EO 13058, the Federal workplace indoor air law, was not exercised by the Bush Administration but should be integral to the proposed health reform agenda of the Obama Administration.

### Implications for Global Tobacco Control

As the implementation of the FCTC proceeds, countries will be implementing an array of tobacco control policies as part of their international commitment. The principle of policy coherence outlined in this paper for the United States may spur countries to think more broadly across government functions about the ways in which the FCTC is implemented. This includes types of government agencies for which tobacco control is a relevant area, potential collaborations across agencies, programmatic and policy content, and infrastructure requirements needed for implementation. It also includes political strategies that may be used to achieve full implementation of the FCTC. A move toward policy coherence on tobacco control in the United States may act as a model for other countries to follow, both in its successes and failures. The material, human capital, and intellectual resources in the U.S. committed to support and that emerge as a result of policy coherence in tobacco control may be leveraged in other countries.

A comprehensive federal agenda on tobacco control will be a critical part of the Obama Administration’s health care reform efforts. After eight years of neglect on the leading cause of death in the United States and a prolonged legislative conundrum over tobacco product and nicotine regulation, a drastic policy change on tobacco control – one geared toward policy *coherence* – is in order. Most urgently, FDA regulatory authority over tobacco, ratification of the FCTC, and a firm resolution to the RICO case must become reality. In order for each of these three critical areas of tobacco control to be adequately addressed and carried out, however, USG agencies must be brought together and empowered to take concerted effort on tobacco control that results in true policy coherence. We believe this change in direction is based on sound science, especially acceptable to the almost 80% of nonsmoking Americans and the 70% of smoking Americans who want to quit, and in the best fiscal and health interests of the United States. It is change that we not only need, but is long overdue.

NOTES

1. Under the Heart Disease, Cancer and Stroke Amendment (Public Law 89-230), appropriations were made to establish “regional cooperative agreements among medical schools, research institutions, and hospitals for research and training… and for related demonstrations of patient care in the fields of heart disease, cancer, stroke, and related diseases.” The Heart Disease, Cancer and Stroke Amendment created 56 Regional Medical Programs to address President Johnson’s charge of reducing the burden of disease from heart disease, cancer and stroke. The Regional Medical Programs were subject to cuts in funding under the Nixon administration, with an end to the Programs in 1976.
2. The Interagency Committee on Smoking and Health (ICSH) was created under Public Law 98-474, the Comprehensive Smoking Education Act. The purpose of the committee, chaired by the Surgeon General, includes advising “the Secretary, HHS, and the Director, CDC, on coordination of research, educational programs, and other activities within the Department that relate to the effect of smoking on human health, and on coordination of these activities with other Federal, State, local, and private agencies.” In practice, the ICSH meets once or twice a year to address one specific topic in tobacco control and take learnings back to members’ home agencies. The ICSH does not have the legislative or fiscal authority to coordinate activities across the federal government or lead a national agenda on tobacco control.
3. Title 42, Chapter 6A, Section 300x-26 of the U.S. Code links the receipt of states’ substance abuse and mental health block grants to the enactment and enforcement of prohibitions limiting the sale of tobacco to individuals under the age of 18 years. The change in code was brought about by Section 1926, Public Law 1002-321, known as the Synar Amendment to the Alcohol, Drug Abuse, and Mental Health Administration Reorganization Act. The regulation requires states to: (a) have in effect a law prohibiting the manufacturer, retailer, or distributor of tobacco products from selling or distributing such products to any individual under the age of 18, (b) enforce such laws in a reasonable manner to reduce the extent to which tobacco products are available to individuals under the age of 18, (c) conduct annual random, unannounced inspections to ensure compliance with the law, and (d) develop a strategy and timeframe for achieving an inspection failure rate of less than 20% of outlets accessible to youth.
4. After withdrawing its Indoor Air Quality proposal in 2001, OSHA’s position is that environmental tobacco smoke contains some of the air contaminants regulated by OSHA, but not tobacco smoke as a whole. Since exposure to contaminants in tobacco smoke that are regulated by OSHA do not exceed permissible exposure limits in normal situations, OSHA does not concern itself with environmental tobacco smoke.
5. Preliminary estimates from the World Health Organization suggest that over 20% of tuberculosis (TB) cases worldwide may be attributable to smoking. Epidemiological data on the excess morbidity and mortality risks of smoking among TB patients suggests a causal link between smoking and adverse TB outcomes. Among TB patients who smoke, those that enter direct observation therapy programs to treat TB should be provided with smoking cessation counseling and treatment each time they interact with a health care provider – be it daily, three times a week, or once a week.

REFERENCES

1. Trust for America's Health (2008) Blueprint for a Healthier America. Washington D.C.

2. Centers for Disease Control and Prevention (1999) Ten Great Public Health Achievements -- United States, 1900-1999. Morbidity and Mortality Weekly Report 48: 241-243.

3. Mokdad AH, Marks, James S., Stroup, Donna F., Gerberding, Julie L. (2004) Actual Causes of Death in the United States, 2000. JAMA 291: 1238-1246.

4. Family Smoking Prevention and Tobacco Control Act (2007) S. 625

5. Givel M (2007) FDA Legislation. Tobacco Control 16: 217-218.

6. Gardiner P (2008) Menthol Moves Center Stage. Tobacco-Related Disease Research Program Newsletter.

7. Centers for Disease Control and Prevention (2002) Cigarette smoking among adults --- United States, 2000. Morbidity and Mortality Weekly Report 51: 642-645.

8. Centers for Disease Control and Prevention (2002) Annual Smoking-Attributable Mortality, Years of Potential Life Lost, and Economic Costs - United States, 1995--1999. Morbidity and Mortality Weekly Report 51: 300-303.

9. The President's Commission on Heart Disease (1964) Report to the President: A National Program to Conquer Heart Disease, Cancer and Stroke, Volume 1. Washington, D.C.: Government Printing Office. 114 p.

10. National Cancer Institute (2008). The National Cancer Act of 1971.

11. The National Cancer Act of 1971. pp. 778-786.

12. National Cancer Institute (2006) NIH, NCI Appropriations, 1997–2007. Journal of the National Cancer Institute 98: 376.

13. Foster S, Niederhausen, Piet (2000) Federal HIV/AIDS Spending. A Budget Chartbook. Menlo Park, CA: The Kaiser Family Foundation. 45 p.

14. Hohmann J (2008) Bush AIDS initiative gets bipartisan renewal. Los Angeles Times. Los Angeles. pp. A-13.

15. U.S. Department of Health and Human Services (2000) Reducing Tobacco Use: A Report of the Surgeon General. Atlanta, GA: U.S. Department of Health and Human Services, Centers for Disease Control and Prevention, National Center for Chronic Disease Prevention and Health Promotion, Office on Smoking and Health.

16. Institute of Medicine (2007) Ending the Tobacco Problem: A Blueprint for the Nation. Washington, D.C.: The National Academies Press.

17. President's Cancer Panel (2007) Annual Report for 2006-2007. Promoting Healthy Lifestyles: Policy, Program, and Personal Recommendations for Reducing Cancer Risk. Bethesda, MD: U.S. Department of Health and Human Services, National Institutes of Health, National Cancer Institute.

18. Centers for Disease Control and Prevention (2007) Smoking & Tobacco Use.

19. U.S. Department of Health and Human Services (1989) Reducing the Health Consequences of Smoking: 25 Years of Progress. A Report of the Surgeon General. U.S. Department of Health and Human Services, Public Health Service, Centers for Disease Control, Center for Chronic Disease Prevention and Health Promotion, Office on Smoking and Health. DHHS Publication No. (CDC) 89-8411 DHHS Publication No. (CDC) 89-8411.

20. Fiore MC, Croyle, Robert T., Curry, Susan J., Cutler, Charles M., Davis, Ronald M, Gordon, Catherine, Healton, Cheryl, Koh, Howard K., Orleans, Tracy, Richling, Dennis, Satcher, David, Seffrin, John, Williams, Christine, Williams, Larry N., Keller, Paula A., Baker, Timothy B. (2004) Preventing 3 million premature deaths and helping 5 million smokers quit: A national action plan for tobacco cessation. American Journal of Public Health 94: 205-210.

21. Campaign for Tobacco-Free Kids AHA, American Cancer Society, American Lung Association (2003) A Broken Promise to Our Children: The 1998 State Tobacco Settlement Five Years Later. 137 p.

22. National Cancer Institute (2005) ASSIST: Shaping the Future of Tobacco Prevention and Control. Bethesda, MD: U.S. Department of Health and Human Services, National Institutes of Health, National Cancer Institute. NIH Pub. No. 05-5645 NIH Pub. No. 05-5645. 576 p.

23. Glantz SA, Balbach, Edith D. (2000) Tobacco Wars. Berkeley and Los Angeles: University of California Press. 469 p.

24. Gross CP, Soffer, Benny, Bach, Peter B., Rajkumar, Rahul, Forman, Howard P. (2002) State expenditures for tobacco-control programs and the tobaccl settlement. New England Journal of Medicine 347: 1080-1086.

25. Schroeder SA (2002) Conflicting Dispatches from the Tobacco Wars. New England Journal of Medicine 347: 1106-1109.

26. Ponder P, Jefferson, Anne-Marie, Backinger, Cathy, Grana, Rachel (2006) Tobacco-related research funding at the National Cancer Institute: Portfolio analysis, fiscal year 2003. Nicotine & Tobacco Research 9: 1053-1057.

27. Gross CP, Anderson, Gerard F., Powe, Neil R., (1999) The relation between funding by the National Institutes of Health and the burden of disease. New England Journal of Medicine 340: 1881-1887.

28. Curry CW, De, Anindya K., Ikeda, Robin M., Thacker, Stephen B. (2006) Health burden and funding at the Centers for Disease Control and Prevention. American Journal of Preventive Medicine 30: 269-276.

29. Centers for Disease Control and Prevention (2006) State Medicaid Coverage of Tobacco-Dependence Treatments - United States, 2005. Morbidity and Mortality Weekly Report 55: 1193-1197.

30. Centers for Medicare and Medicaid Services (2007) Smoking Cessation - Overview.

31. Indian Health Service (2006) Tobacco Control Strategic Plan. 20 p.

32. Centers for Disease Control and Prevention (2004) Prevalence of Cigarette Use Among 14 Racial/Ethnic Populations - United States, 1999-2001. Morbidity and Mortality Weekly Report 53: 49-52.

33. Berg AO (2003) Counseling to Prevent Tobacco Use and Tobacco-Caused Disease. Recommendation Statement. Agency for Healthcare Research and Quality. AHRQ Pub. No. 04–0526 AHRQ Pub. No. 04–0526. 5 p.

34. Task Force on Community Preventive Services (2005) Tobacco. In: Zaza S, Briss, Peter A., Harris, Kate W., editor. The Guide to Community Preventive Services. New York: Oxford University press. pp. 3-79.

35. Fiore MC, Jaen, Carlos Roberto, Baker, Timothy B., et. al. (2008) Treating Tobacco Use and Dependence: 2008 Update. Rockville, MD: U.S. Department of Health and Human Services, Public Health Service.

36. Substance Abuse and Mental Health Services Administration (2007) Youth Tobacco Sales. Rockville, MD: U.S. Department of Health and Human Services, Substance Abuse and Mental Health Services Administration. DHHS Publication No. (SMA) 07-4300 DHHS Publication No. (SMA) 07-4300. 8 p.

37. Slade J, Connolly, Gregory N., Davis, Ronald M., Douglas, Clifford E., Henningfield, Jack E., Hughes, John R., Kozlowski, Lynn T., Myers, Matthew L. (1992) Report of the Tobacco Policy Research Study Group on Tobacco Products. Tobacco Control 1: S4-S9.

38. US Code Title 15 Chapter 36.

39. Federal Trade Commission (2008) Proposal to Rescind FTC Guidance Concerning the Current Cigarette Test Method. pp. 9.

40. U.S. General Accounting Office (2004) Cigarette smuggling: Federal law encorcement efforts and seizures increasing. Wasington, D.C.: U.S. General Accounting Office.

41. U.S. Environmental Protection Agency (1992) Respiratory Health Effects Of Passive Smoking: Lung Cancer and Other Disorders. Washington, D.C.

42. United States Department of Agriculture Economic Research Service, Tobacco Outlook.

43. Waxman HA, Durbin, Richard J. (2003) Memorandum to the President on tobacco control. In: President T, editor. Washington D.C.

44. U.S. Government Accounting Office (1990) Trade and Health Issues. Dichotomy Between U.S. Tobacco Export Policy and Antismoking Initiatives. Washington, D.C.

45. Wilkenfield JP (2003) U.S. Should Protect Public Health and Exclude Tobacco From Proposed Free Trade Area of the Americas. Washington, D.C.: Campaign for Tobacco-Free Kids.

46. World Health Organization (2003) WHO Framework Convention on Tobacco Control. Geneva, Switzerland.

47. United States of America v. Philip Morris Incorporated, et. al. (2006)

48. Partnership for Prevention (2008) The Need for Prevention-Centered Health Reform. Washington D.C.: Partnership for Prevention.

49. Curry SJ, Orleans, Tracy, Keller, Paula, Fiore, Michael (2006) Promoting smoking cessation in the healthcare environment. American Journal of Preventive Medicine 31: 269-272.

50. Lightwood JM, Dinno, Alexis, Glantz, Stanton A. (2008) Effect of the California Tobacco Control Program on personal health care expenditures. PLoS Medicine 5: e178.

51. American Legacy Foundation (2007) Saving lives, saving money II: Tobacco-free states spend less on Medicaid. Washington, D.C.: American Legacy Foundation.

52. Fichtenberg CM, Glantz, Stanton A. (2000) Association of the California Tobacco Control Program with declines in cigarette consumption and mortality from heart disease. New England Journal of Medicine 343: 1772-1777.

53. Centers for Disease Control and Prevention (1996) Cigarette smoking before and after an excise tax Increase and an antismoking campaign -- Massachusetts, 1990-1996. Morbidity and Mortality Weekly Report 45: 966-970.

54. American's For Non-Smokers Rights Foundation (2008) Percent of U.S. State Populations Covered by 100% Smokefree Air Laws. pp. 2.

55. Everett Jones S, Fisher, Carolyn J., Greene, Brenda Z., Hertz, Marci F., Pritzi, Jane, (2007) Healthy and safe school environment, part I: Results from the School Health Policies and Programs Study 2006. Journal of School Health 77: 522-543.

56. Pro-Children Act of 1994.

57. Department of Defense Directive 1010.15

58. Department of Defense Instruction 1010.15

59. Smith EA, Blackman, Virginia S., Malone, Ruth E. (2007) Death at a Discount: How the Tobacco Industry Thwarted Tobacco Control Policies in U.S. Military Commissaries. Tobacco Control 16: 38-46.

60. Kessler D (2000) A question of intent: A great American battle with a deadly industry. New York: Public Affairs. 492 p.

61. Gilhooley M (2002) Tobacco unregulated: Why the FDA failed, and what to do now. The Yale Law Journal 111: 1179-1213.

62. Federal Trade Commission Act. United States Code Title 15 Section 45.

63. National Cancer Institute (2008) The Role of the Media in Promoting and Reducing Tobacco Use. Tobacco Control Monograph No. 19. Bethesda, MD: U.S. Department of Health and Human Services, National Institutes of Health, National Cancer Institute.

64. Landman A, Ling, Pamela M., Glantz, Stanton A. (2002) Tobacco Industry Youth Smoking Prevention Programs: Protecting the Industry and Hurting Tobacco Control. American Journal of Public Health 92: 917-930.

65. National Working Group for ACTTION (2008) A Call for ACTTION: Access to Cessation Treatment for Tobacco in Our Nation. Washington D.C.: Partnership for Prevention.

66. Wilson M (2008) Prevalence of tobacco abuse in a United States Marine Corp Infantry Battalion Forward deployed in the Haditha Triad Area of Operations, Al Anbar, Iraq. CHEST 134: s53001.

67. Centers for Disease Control and Prevention (2008) Smoking-Attributable Mortality, Years of Potential Life Lost, and Productivity Losses — United States, 2000–2004. Morbidity and Mortality Weekly Report 57: 1226-1227.

68. Womach J (2005) Tobacco price support: An overview of the program. Washington D.C.: Congressional Research Service. 6 p.

69. U.S. Department of Agriculture (2009) Tobacco Transition Payment Program.

70. Womach J (2006) Tobacco-related programs and activities of the U.S. Department of Agriculture: Operation and cost. Washington, D.C.: Congressional Research Service. 5 p.

71. U. S. General Accounting Office (2003) Pesticides on tobacco: Federal activities to assess risks and monitor residues. Washington, D.C.: U.S. General Accounting Office.

72. Daynard R, Bates, Clive, Francey, Neil (2000) Tobacco litigation worldwide. British Medical Journal 320: 111-113.

73. Tobacco Research Implementation Group (1998) Tobacco Research Implementation Plan: Priorities for Tobacco Research Beyond the Year 2000. The National Cancer Institute. 63 p.

74. Delnevo CD, Bauer, Ursula E. (2008) Monitoring the tobacco use epidemic III. The host: data source and methodological challenges. Preventive Medicine doi:10.016/jypmed.2008.09.008.

75. Cruz TB (2008) Monitoring the tobacco use epidemic IV. The vector: Tobacco industry data sources and recommendations for research and evaluation. Preventive Medicine doi:10.1016/j.ypmed.2008.10.002.

76. Baba A, Cook, Daniel M., McGarity, Thomas O., Bero, Lisa A. (2005) Legislating “Sound Science”: The Role of the Tobacco Industry. American Journal of Public Health 95: S20-S27.

77. Mathers CD, Loncar, Dejan (2006) Projections of global mortality and burden of disease from 2003 to 2030. PLoS Medicine 3: e442.

78. Mackay J, Eriksen, Michael (2002) The Tobacco Atlas. Geneva, Switzerland: World Health Organization.

79. U.S. Government Accounting Office (2003) Tobacco Exports: USDA's Foreign Agricultural Service Lacks Specific Guidance for Congressional Restrictions on Promoting Tobacco. Washington, D.C. 23 p.

80. World Health Organization WTO (2002) WTO Agreements & Public Health.

81. Weissman R (2003) International Trade Agreements and Tobacco Control: Threats to Public Health and the Case for Excluding Tobacco From Trade Agreements. Washington, D.C.: Essential Action. 28 p.

82. Shibuya K, Ciecierski, Christina, Guindon, Emmanuel, Bettcher, Douglas W., Evans, David B., Murray CJL (2003) WHO Framework Convention on Tobacco Control: development of an evidence based global public health treaty. British Medical Journal 327: 154-157.

83. Beelman M, Davidson, Zoe (1999) Support for Tobacco Trade: Up in Smoke?

84. U.S. Agency for International Development (2006) Tobacco Policy. ADS Chapter 210.

85. United States Codes - Highways. pp. 125.

ACKNOWLEDGMENTS

None.

ABBREVIATIONS

AHRQ, Agency for Healthcare Research and Quality; ATF, Bureau of Alcohol, Tobacco, Firearms and Explosives; CDC, Centers for Disease Control and Prevention; CMS, Centers for Medicare and Medicaid Services; DHHS, Department of Health and Human Services; DOD, Department of Defense; DOJ, Department of Justice; DOS, Department of State; DPC, Domestic Policy Council; EO, Executive Order; EPA, Environmental Protection Agency; FCTC, Framework Convention on Tobacco Control; FDA, Food and Drug Administration; FTC, Federal Trade Commission; ICSH, Interagency Committee on Smoking and Health; IHS, Indian Health Service; IOM, Institute of Medicine; MCH, maternal and child health; MSA, Master Settlement Agreement; NA/AN, Native American/Alaska Native; NCI, National Cancer Institute; NIH, National Institutes of Health; OMB, Office of Management and Budget; OSH, Office on Smoking and Health; OSHA, Occupational Safety and Health Administration; PACHA, President’s Advisory Council on HIV and AIDS; PEPFAR, President’s Emergency Plan For AIDS Relief; RICO, Racketeer Influence and Corrupt Organizations Act; SAMHSA, Substance Abuse and Mental Health Services Administration; SCHIP, State Children’s Health Insurance Program; SHS, second hand smoke; USAID, U.S. Agency for International Development; USDA, U.S. Department of Agriculture; USG, United States Government; USTR, United States Trade Representative; VA, U.S. Department of Veterans Affairs; WHO, World Health Organization.

Table 1. Health and economic burdens of smoking, United States.

Tobacco use…

**…is the leading preventable cause of death**. At least 443,000 annual premature deaths in the United States from 2000-2004 were attributable to smoking [67].

**…leads to premature death.** During 2000-2004, 5.1 million years of productive life were lost due to cigarette smoking and exposure to passive smoking per year [67].

**…contributes to health disparities.** African-Americans, Native Americans/Alaska Natives, the poor, and people with lower educational attainment suffer from a higher burden of disease and disability from smoking.

…is a major cause of cancer in the lung, larynx, pharynx, mouth, bladders. It also causes cancer in the pancreas, cervix, kidney, and stomach.

…causes deaths from heart disease, stroke, chronic obstructive pulmonary disease.

**…is a fiscal burden.** Cigarette smoking and exposure to tobacco smoke results in productivity losses of approximately $97 billion annually and approximately $96 billion in annual U.S. medical expenditures [67].

.Figure 1. Department of Health and Human Services Organizational Chart, November 2008.


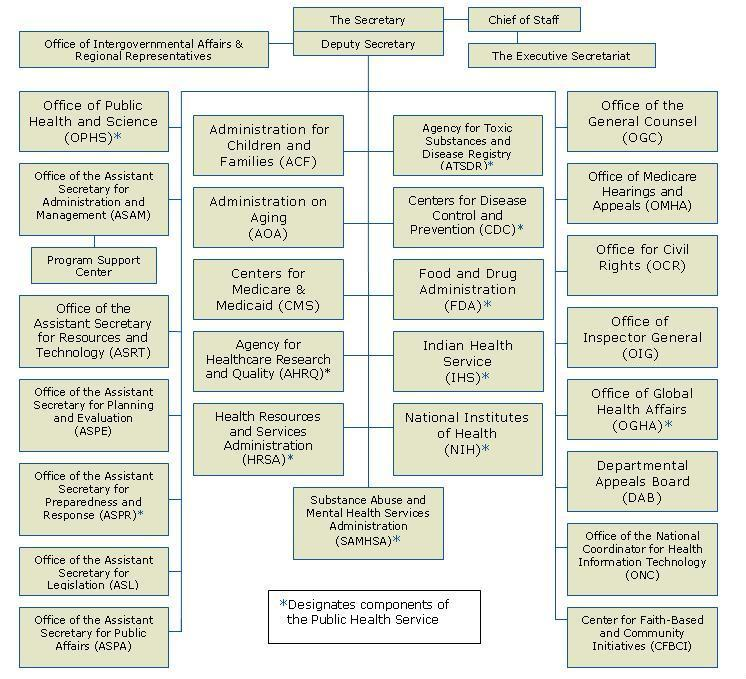

Supplement: Text S1 — Longer, more comprehensive version of the article. (0.65 MB DOC) [file pmed.1000079.s001.doc]
